# Advances in protein ubiquitination modification and immune evasion of breast cancer

**DOI:** 10.3389/fcell.2026.1795272

**Published:** 2026-03-16

**Authors:** Yifan Wu, Jiangdong Jin, Xin Cheng, Ziyi Fu, Hui Xie

**Affiliations:** 1 Department of Breast Surgery, The First Affiliated Hospital with Nanjing Medical University, Nanjing, China; 2 Nanjing Medical University, Nanjing, China; 3 Department of Breast Surgery, Jiangsu Cancer Hospital with Nanjing Medical University, Nanjing, China

**Keywords:** breast cancer, deubiquitinating enzymes (DUBs), immunotherapy, protein ubiquitination modification, tumor immune evasion

## Abstract

Protein ubiquitination is one type of post-translational modification that can alter many properties of the protein—stability, activity, subcellular location, binding affinity for other proteins, etc. It has been involved in almost all life activities, including immune response regulation, DNA damage repair, cell cycle regulation, cell proliferation, apoptosis, and protein degradation. Moreover, it is associated with many kinds of disease, such as neurodegenerative diseases, various tumors, immune diseases, and metabolic diseases. Recent reports revealed that protein ubiquitination plays a key role in the breast cancer (BC) immune evasion. “Immune evasion” refers to the ability of tumor cells/pathogens to evade recognition and attack by the immune system through different mechanisms; it includes three interconnected processes: “immune editing,” “antigenic variation,” and “immunosuppressive molecules expression.” These three points lead to the difficulty for the body to clear the transformed cells/pathogens promptly. Here, we summarize the mechanisms of protein ubiquitination in breast cancer immune evasion and explore new strategies targeting protein ubiquitination to combat BC.

## 1 Introduction

According to the latest data released by IARC/WHO, BC is the highest-incidence malignant tumor in the world and threatens the physical and mental health of women. It is estimated that by 2050, the number of global BC patients will have increased by 38%, and more than 1.1 million people will die each year (Migliaccio et al., 2024). Based on this forecast, it seems that the prevention and treatment of BC still face a significant challenge globally. BC ranks first among female malignancies in my country. According to statistics in 2022, there were 357,200 new cases of BC in China, and the average incidence rate is about 33 per 100,000 women. In view of the background that the pace of urbanization is increasing, age at first childbirth and weight are increasing due to changes in dietary structure and living environments in modern society. The occurrence risk of BC is quietly rising. Male BC is rare, accounting for less than one percent of all BCs (<1%) (Migliaccio et al., 2024; Pederson et al., 2024). From the point of view of cell biology, BC can be further divided into HR (+), HER2 (+), or TNBC based on the expression status of relevant proteins, and into ILC and IDC based on differences in origin sites. Different subtypes usually need different treatments (Pantazi et al., 2024; Zeng et al., 2025a). One of the key responsibilities of the human immune system is to identify and eliminate cells that have become cancerous. But some early-stage cancer cells successfully evade immune surveillance and continue to grow into large tumors—known as “immune escape”. The concept of BC immune escape refers to various approaches taken by tumors to prevent them from being detected and eliminated by the immune system (Wang H. et al., 2025); mainly manifested as downregulation of the expression levels of TAA (tumor-associated antigen), interference with the activity of effector immune cells, activation of immunosuppression-related signaling pathways, and so on. The complex mechanisms and heterogeneity of clinical manifestations pose significant challenges for treating and preventing diseases. As the global burden of BC continues to rise, it is urgent to understand its characteristics from multiple perspectives and propose effective intervention measures.

Protein, as the primary executor of biological function, plays a vital role in maintaining cell homeostasis. Changes in protein levels can cause various pathologies in protein synthesis, folding, transport, or other biological processes. Protein degradation mainly occurs through two pathways in human cells: the ubiquitin–proteasome system (UPS) and lysosome–autophagy pathway; among which, UPS accounts for approximately 80%–90% of total protein degradation (Alhasan et al., 2024). Ubiquitination is a type of post-translational modification (PTM), a covalent attachment of ubiquitin (a small regulatory protein). There are seven lysines (K6, K11, K27, K29, K33, K48, K63) and one Met (Met1) (Wang G. et al., 2024). It is under the joint action of three enzymes: ubiquitin-activating enzyme (E1), ubiquitin-conjugating enzyme (E2), and ubiquitin ligase (E3) (Huang et al., 2024). Ubiquitination regulates protein stability, activity, location, interaction with macromolecules, etc., and participates in almost all aspects of cellular physiological activities, including immune regulation (Ch et al., 2024), DNA damage repair (Fu et al., 2024), cell cycle (Zhang et al., 2025), cell proliferation (Wang T. et al., 2024), apoptosis (Agrata and Komander, 2025), and protein degradation (Fiil and Gyrd-Hansen, 2014), and so on. Therefore, it is closely related to many diseases, including neurodegenerative diseases (Ch et al., 2024), tumors (Alhasan et al., 2024), immune diseases (Fu et al., 2024), and metabolic diseases (Zhang et al., 2025). In this article, we will review how protein ubiquitination modification is related to immune evasion in BC and provide some ideas and new targets for BC treatment.

**FIGURE 1:**
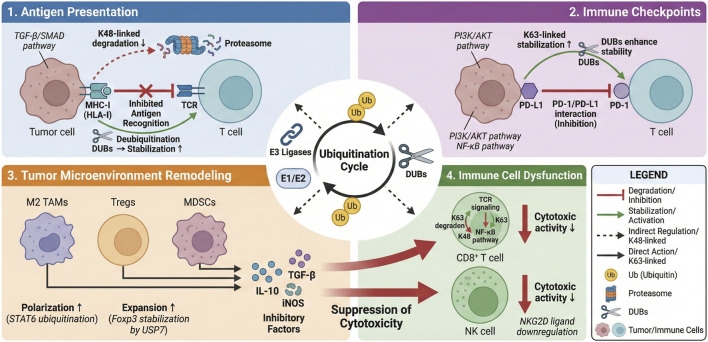
The Ubiquitin Switch: Orchestrating Immune Evasion in Breast Cancer: This graphical abstract illustrates the central role of the protein ubiquitination system in modulating multiple axes of immune evasion in breast cancer (BC). The dynamic balance between ubiquitin ligases (E3s) and deubiquitinases (DUBs) acts as a master regulator, fine-tuning the stability and function of key substrates. This regulation impacts: (1) Antigen Presentation by targeting MHC-I molecules for degradation; (2) Immune Checkpoint Expression by stabilizing PD-L1; (3) Tumor Microenvironment by promoting the polarization and function of immunosuppressive cells (M2 TAMs, Tregs, MDSCs); and (4) Immune Cell Function by impairing cytotoxic activity. The concerted effect of these pathways facilitates immune evasion and tumor progression. Targeting this system with specific inhibitors (e.g., USP inhibitors, E3 ligase inhibitors) or degraders (PROTACs) represents a promising therapeutic strategy to restore anti-tumor immunity in BC.

Having outlined the clinical significance of immune evasion in BC and introduced ubiquitination as a key regulatory PTM, we now delve into the fundamentals of the ubiquitin system itself. A precise understanding of its components, mechanisms, and functional diversity is essential to appreciate how it orchestrates immune evasion, which will be detailed in subsequent sections (Figure 1).

## 2 Protein ubiquitination

### 2.1 Ubiquitination overview

Ubiquitin is a small, molecular-weight protein, widely distributed in eukaryotic cells, consisting of 76 amino acids, and there are seven lysine sites (K6, K11, K27, K29, K33, K48, and K63) and the first methionine site at the N terminus (M1) (Wang G. et al., 2024). Based on the connection method, it can be divided into monoubiquitin chains and polyubiquitin chains. The process of linking one or more ubiquitin molecules with covalent bonds to another protein molecule under the action of a group of enzymes is called ubiquitination modification. The enzymes mainly involved in protein ubiquitination include ubiquitin-activating enzyme (E1), ubiquitin-conjugating enzyme (E2), and ubiquitin ligase (E3) (Huang et al., 2024). Firstly, E1 uses ATP energy to catalyze the reaction between its own cysteine residue thiol and the carboxyl group at the C-terminus of ubiquitin to form a high-energy thioester bond, thereby generating Ub-E1 enzyme complexes, which activate free ubiquitin molecules; secondly, Ub-E1 enzyme complexes transfer ubiquitin to E2 through a transfer reaction to form the Ub-E2 enzyme complex; finally, Ub-E2 enzyme complexes transfer ubiquitin to the target protein. According to the classification of E3 enzymes, it can be further divided into two pathways: in one pathway, E3 enzymes recognize targets specifically and directly couple the C-terminus of ubiquitin to the ε-amino group of the lysine residues on the target protein; in another path, ubiquitin is first transferred to E3 enzymes to form a thioester intermediate before being transferred onto the substrate protein (Huang et al., 2024; Wang T. et al., 2024). Therefore, through this series of enzymatic reactions, ubiquitin can be correctly conjugated to the substrate protein, and the E3 ubiquitin ligase selectively recognizes substrates for ubiquitination.

### 2.2 Ubiquitination functions

Based on which ubiquitin residue is connected to a protein, it could be divided into M1, K6, K11, K27, K29, K33, K48, and K63 ubiquitination modifications (M1-, K6-, K11-, K27-, K29-, K33-, K48-, and K63-linked polyubiquitination modifications) (Wang G. et al., 2024). Based on previous research, different ubiquitination modifications are involved in distinct cellular processes (Agrata and Komander, 2025). Among them, M1-linked linear polyubiquitin chains mainly play a role in protein kinase activation and NF-κB signaling pathway activation (Fiil and Gyrd-Hansen, 2014); K6-linked polyubiquitin chains may be related to some aspects of immune regulation and so on (Yang et al., 2023); K11-linked polyubiquitin chains not only regulate cell cycle progression (Zhang S. et al., 2023), but also form K48/K11 branched chains with K48-linked chains to promote the degradation of proteins by the proteasome (Lopata et al., 2020); both K27-linked chains and K29-linked chains are related to autophagy or DNA damage response (Gatti et al., 2015); most K33-linked polyubiquitin chains are related to autophagy (Sun et al., 2024); K48-linked polyubiquitin chains mainly play a regulatory role in protein stability and promote the degradation of modified proteins (Tang et al., 2020); K63-linked polyubiquitin chains are primarily involved in transcription activation, autophagy, and DNA damage response processes (Lim and Lim, 2011) (Table 1).

**TABLE 1:** The functions of different ubiquitin residue linkage sites on proteins.

Types	Function	References
M1	Activating nuclear factor-κB (NF-κB) and mitogen-activated protein kinases (MAPKs)	Doi: 10.1111/febs.12944
	Emerged as essential signalling scaffolds that regulate pro-inflammatory responses, anti-viral interferon responses, cell death and xenophagy of bacterial pathogens downstream of innate immune receptors	Doi: 10.1038/s41418-020-00702-x
K6	Activating STING during antiviral responses	Doi: 10.15252/embr.202357528
	Chromatin loading	Doi: 10.1093/nar/gkac011
	Inducing phase separation to trigger purinosome assembly for enhancing DNPS pathway flux	Doi: 10.1016/j.molcel.2023.09.028
	Regulating myocardial oxidative stress, cardiomyocyte apoptosis and mitochondrial fragmentation	Doi: 10.1007/s00018-024-05257-5
	Participating in the cellular and tissue toxicity of reactive aldehydes	Doi: 10.1016/j.molcel.2023.10.011
	Activating ERK signaling pathway	Doi: 10.1002/1878-0261.13290
	Promoting ferroptosis susceptibility	Doi: 10.1016/j.redox.2024.103350
	Promoting lipid droplet formation and fatty acid β-oxidation	Doi: 10.1038/s41419-024-07054-1
K11	Enhancing the cellular level of ROS and lipid peroxidation	Doi: 10.1038/s41418-023-01239-5
	Positively correlated with the lymphatic metastasis status, high tumor stage, histological grade, and poor prognosis of BCa patients	Doi: 10.1038/s41419-023-05938-2
	A signal for degradation by the proteasome	Doi: 10.3390/ijms21155369
	Inhibiting the post-endoplasmic reticulum (ER) trafficking of TLRs and TLRs-mediated immune responses	Doi: 10.1002/advs.202105391
	Activating NF-κB signaling pathway	Doi: 10.1016/j.ijbiomac.2024.134426
	The functional activation of YAP/TAZ and hippo pathway	Doi: 10.1038/s41419-023-05777-1
	Regulating cell cycle progression and autophagy	Doi: 10.1172/JCI162434
	The regulation of the inflammatory innate immune response	Doi: 10.1038/s41418-021-00803-1
	Regulating the substrates of the anaphase-promoting complex and control progression through mitosis	Doi: 10.1016/j.tcb.2011.08.008
K27	Immune regulation	Doi: 10.1002/JLB.4RU0620-397RR
	Cardiac hypertrophy and failure	Doi: 10.1161/CIRCULATIONAHA.121.054827
	NLRP3-NEK7 complex formation and NLRP3 oligomerization	Doi: 10.15252/embj.2023113481
	Selective autophagy to evade host antiviral immunity	Doi: 10.1080/15548627.2022.2139921
	Elevation of the MEK/ERK signaling	Doi: 10.1038/s41467-019-09844-0
	Macroautophagy/autophagy	Doi: 10.1080/15548627.2023.2293442
	Autoimmunity	Doi: 10.1186/s12929-025-01120-2
	The activation of MAVS and STING	Doi: 10.1128/jvi.01513-23
	DNA damage response (DDR)	Doi: 10.1016/j.celrep.2014.12.021
	cGAS-mediated type I interferonopathies	Doi: 10.1016/j.celrep.2024.114248
K29	Promoting antiviral innate immunity	Doi: 10.1038/s41467-021-21456-1
	Normal viral cycle progression and for maximal virion production	Doi: 10.1128/mBio.00305-20
	Small-molecule-induced degradation	Doi: 10.1016/j.molcel.2021.01.023
	Regulating proteotoxic stress response and cell cycle	Doi: 10.1038/s41589-021-00823-5
	Activating cGAS/STING	Doi: 10.1038/s41467-023-38784-z
​	Ribosome biogenesis	Doi: 10.1016/j.molcel.2024.05.018
	Balancing proliferation and invasion	Doi: 10.1016/j.canlet.2025.217624
	Regulating the RLR signaling pathway	Doi: 10.4049/jimmunol.2200544
	Regulating autophagy, proteostasis and liver metabolism	Doi: 10.1038/s41467-021-21715-1
K33	Autophagy	Doi: 10.1080/15548627.2023.2299514
	Complishment of active DNA demethylation	Doi: 10.1016/j.molcel.2021.05.022
	Regulating PKM2 polymerization	Doi: 10.1161/CIRCRESAHA.124.325049
	NLRP3 inflammasome activation and coagulation	Doi: 10.1038/s41419-024-06991-1
	Promoting RNA virus replication and virus-induced inflammation	Doi: 10.1038/s41419-023-05923-9
	PI3K-Akt pathway	Doi: 10.1111/imm.13787
	Furin anterograde transport	Doi: 10.1002/advs.202403732
	Fatty acid synthesis	Doi: 10.1038/s41388-024-03056-7
	Affecting cisplatin resistance and cancer metastasis	Doi: 10.1016/j.drup.2024.101096
K48	Autophagy	Doi: 10.1080/15548627.2021.1912270
	Ferroptosis and radiosensitization	Doi: 10.1038/s41418-022-01051-7
	NLRP3 inflammasome	Doi: 10.1084/jem.20182091
	STING activation	Doi: 10.1016/j.celrep.2024.114135
	IFN immune balance	Doi: 10.4049/jimmunol.2100125
K63	DNA repair, endocytosis and NFκB signaling	Doi: 10.1016/j.nbd.2010.08.001
	Virus entry, tropism and pathogenesis	Doi: 10.1038/s41586-020-2457-8
	Senescence	Doi: 10.3390/cells11193115
	Activating the RIG-I-MAVS signaling cascade	Doi: 10.1038/s41423-023-01065-2
	ERK signalling pathway	Doi: 10.1038/s41556-021-00732-8

### 2.3 Key enzymes in ubiquitination

No E1 enzyme has been found in human cells to date, but two have been reported to be involved in Ub conjugation (UBA1 and UBA6). There were about 40 kinds of E2 enzymes and over 600 kinds of E3 enzymes found in human cells thus far. The name implied that E3 ubiquitin ligase could recognize substrates in a specific way, and therefore became the main object to study nowadays. According to different E2-binding domains and mechanisms of Ub transfer reactions, they could be roughly divided into three categories: RING finger-E3 (about 600 kinds), HECT (homologous to the E6AP C-terminus) domain-E3 (28 types), and RBR (between RING and RING) domain-E3 (14 types). Compared with RING-E3, which transfers Ub directly from E2 to substrates, both HECT and RBR E3s transferred Ub to substrates through a two-step reaction (Kochańczyk et al., 2024). Like other PTMs (post-translational modifications), ubiquitination is reversible. DUB (deubiquitinating enzymes) could remove ubiquitin from substrate proteins, modify polyubiquitin chains, and process ubiquitin precursors. Up until now, over 100 DUBs have been identified in human cells, which might be classified into six families based on similarities in primary sequences and domain organizations, namely, USPs (Ub-specific processing proteases), UCHs (Ub C-terminal hydrolases), MJDs (Machado–Joseph domain proteases), OTUs (ovarian tumor proteases), MINDYs (motif interacting with a Novel DUB Family Containing Ubiquitin) [UBIQ], and JAMMs (JAB1/MPN/MOV34 metalloenzyme) (Vogel and Isono, 2024). Typically, E3 ubiquitin ligases increase the ubiquitination of target proteins (substrates), while DUBs decrease the ubiquitination of proteins. Therefore, we often describe the regulatory mechanism of protein ubiquitination as a “dynamic balance.” Taking “normal” condition as reference (“balanced”), the deviation of this balance between ubiquitin ligase/DUB would lead to changes in the ubiquitinated levels of target proteins (substrates), resulting in their degradation by the proteasome (Dawson et al., 2025). Equipped with an understanding of the ubiquitin machinery, we next map the landscape it seeks to control: the multifaceted mechanisms of immune evasion in BC. This chapter will dissect how tumor cells directly evade immune surveillance and remodel the tumor microenvironment (TME) to suppress immunity, setting the stage for exploring ubiquitin’s role in each of these processes (Table 2).

**TABLE 2:** Key immune-regulatory functions of distinct ubiquitin linkage types.

Linkage type	Key functions in immune regulation	Involved process
M1	Activates NF-κB and MAPK signaling pathways: Serves as crucial signaling scaffolds downstream of innate immune receptors (e.g., TNFR, TLRs), regulating pro-inflammatory responses and anti-viral interferon responses	Innate immune signaling, inflammatory response
K6	Activates the STING pathway: Involved in anti-viral immune responses, regulating type I interferon production	Cytosolic DNA sensing, anti-viral innate immunity
K11	Regulates immune cell cycle and proliferation: Can form K48/K11-branched chains to cooperatively promote proteasomal degradation of target proteins, potentially affecting immune cell activation and proliferation	Cell cycle progression, proteasomal degradation
K27	Modulates inflammasome activity and immune responses: Involved in NLRP3 inflammasome activation; can serve as a signal for proteasomal degradation; also participates in negative regulation of TLR trafficking and immune responses	NLRP3 inflammasome activation, TLR signaling modulation, immune feedback
K29	Participates in anti-viral innate immunity: Reported to regulate the RLR (RIG-I-like receptor) signaling pathway, influencing anti-viral responses	Anti-viral innate immunity, RLR signaling pathway
K33	Regulates T-cell receptor signaling and metabolism: Involved in negative regulation of T-cell receptor (TCR) signaling; also associated with metabolic pathways such as PI3K-Akt, potentially impacting immune cell function	TCR signal transduction, immune cell metabolism
K48	Dominates protein degradation, regulating homeostasis of immune signaling proteins: The primary signal for proteasomal degradation. Precisely controls numerous immune-related pathways (e.g., NF-κB, apoptosis, cell cycle) by degrading key signaling proteins (e.g., IκB, pro-apoptotic proteins) or regulatory proteins (e.g., immune checkpoint molecules)	Protein stability, NF-κB pathway, immune checkpoint protein turnover
K63	Dominates non-degradative signal transduction, broadly involved in immune regulation: Acts as a central signaling scaffold in innate immunity (TLR/RLR signaling), inflammatory responses, DNA damage response, and autophagy; also participates in adaptive immune processes such as T-cell activation and B-cell development	TLR/NF-κB signaling, DNA damage repair, autophagy, lymphocyte activation

## 3 Mechanisms of tumor immune escape in BC

The immune system protects its host by removing transformed neoplastic cells as they arise during carcinogenesis; however, neoplastic cells often develop mechanisms of immune resistance that limit both innate and adaptive anti-tumor immunity (Wang H. et al., 2025), which have been demonstrated as key regulators of tumorigenesis and responses to immunotherapy in cancer patients (Wang H. et al., 2025). Immune evasion from tumors is mainly manifested in two aspects: (1) direct avoidance by the tumor, such as upregulation of checkpoint immunoinhibitors, reduction of immunogenicity, loss or reduction of antigen expression, and so on (Kiflu, 2024; Vadakekolathu and Rutella, 2024); (2) indirect immune inhibition induced within the TME through the recruitment or expansion of immunosuppressive cells (Kiflu, 2024; Vadakekolathu and Rutella, 2024), etc. Further study of these mechanisms may bring about newer and more effective therapies. Here, we will introduce the central immune escape mechanisms(s) reported thus far for BC.

### 3.1 Direct evasion of immune surveillance by tumor cells

#### 3.1.1 Upregulation of immune checkpoints

Immune Escape in BC: The term “immune escape” in BC refers to mechanisms by which the tumor can evade immune cell recognition and destruction, primarily through immune checkpoint(s). As an important regulator of the strength/duration of immune responses, so they will not excessively react against damage caused to self-tissues (Shu et al., 2024), the immune checkpoint has instead been co-opted by the neoplastic clone via upregulation of certain immunoinhibitory molecules such as PD-L1/CTLA-4/LAG-3/TIM-3/TIGIT, etc., which suppress the effector functions of immune cells in terms of transcriptional regulation/post-translational modification/signaling pathway modification to confer “immunity” against attack from them (Shi et al., 2024; Wang J. et al., 2024). Very recently, it was reported by (Fang et al., 2021) that progranulin (PGRN), one type of pleiotropic growth factor, may promote breast tumor immune escape via enhancing the interaction between PD-1/PD-L1. Meanwhile, (Wan et al., 2022) reported that PD-L1 may be a direct target gene of METTL3-methylated mRNAs in BC cells: METTL3 increased the abundance of PD-L1 transcripts in an m6A-dependent manner via IGF2BP3.

#### 3.1.2 Decreased immunogenicity of tumor cells

From early research results, we know That Tumors induced by oncogenic viruses have the highest immunogenicity, chemically induced tumors have relatively lower immunogenicity, and spontaneously occurring tumors have the lowest immunogenicity. Due to the difference in immunogenicity of tumors, highly immunogenic tumor cells could be identified by immune cells, stimulating a strong anti-tumor immunity response, being killed, and being eliminated. Still, low immunogenicity tumor cells could hardly be identified and removed by immune cells. After repeated ‘immunological screening’ of tumors by the body, the immunogenicity of surviving tumors would be reduced successively (Savage et al., 2024; Naji et al., 2024).

#### 3.1.3 Antigen expression loss or downregulation

Changes in HLA-I expression, upregulation of PD-L1 expression, and secretion of immunosuppressive cytokines were observed in cancer cells. For cancer cells, HLA-I complexes have been reported to be reduced or even eliminated from cell surfaces to evade immune responses (Bonté et al., 2022). Actually, alteration in HLA-I phenotype is also a common phenomenon in patients with invasive BC. Alterations in HLA-I mainly involve two aspects: irreversible structural abnormalities and reversible regulatory abnormalities. Firstly, irreversible HLA-I abnormalities are primarily caused by loss of heterozygosity (LOH) of chromosome 6 (containing HLA-A/B/C) and chromosome 15 (encoding β2-microglobulin) (Han et al., 2022). Both are frequently seen in BC. Secondly, another potential mechanism for downregulation of HLA-I may involve DNA methylation-mediated silencing of the HLA-I heavy chain gene, the β2-microglobulin gene, and molecules involved in APM (Wickenhauser et al., 2021). Regardless of whether HLA-I is affected from these two perspectives, it can be restored through various intervention measures, such as IFN-γ treatment or other cytokines released after immunotherapy (Zhang C. et al., 2023). Usually, PD-L1 overexpression occurs on the surface of cancer cells. As such, the surrounding T cells would be deactivated by cancer cells, leading to the blockage of anti-tumor immune response (Franken et al., 2024). It has indeed been reported that high-level expression of PD-L1 was observed in specific subsets of BCs (such as small-cell carcinoma of the breast, basal-like subtype, and inflammatory BC) (Vranic et al., 2021).

### 3.2 Alterations in the tumor microenvironment

The TME (tumor microenvironment): the living environment of tumor cells consists of many parts: tumor cells, immune cells, inflammatory factors, extracellular matrix, etc., with “low oxygen, acid, high interstitial fluid pressure” and other features of physical and chemical properties, it is a kind of complex system (Elhanani et al., 2023; Zeng et al., 2025b). The TME is regulated by tumor cells for cytokine secretion and the accumulation of metabolic by-products, which will inhibit the activity and metabolism of immune cells and lead to immunosuppression (Ding et al., 2024). In the TME, there is an increase of TAMs (tumor-associated macrophages), which are often converted into pro-tumor M2 types in response to the anti-inflammatory cytokines (TGF-β1, IL-4, IL-10, IL-13) secreted by Th2 cells. M2 TAMs can inhibit the effects of effector immune cells and promote immune evasion (Wang et al., 2022). In addition, immunosuppressive cells (Tregs, MDSCs), etc., also regulate antigen presentation and recognition, thereby promoting the occurrence of tumor immune evasion (Korbecki et al., 2020; Vanden Abeele and Salzet, 2025).

#### 3.2.1 Immunosuppressive cells

The most recent data from clinical and experimental studies have shown that tumors arise as a consequence of interactions between cancer cells and different types of stromal cells: immune cells, fibroblasts, vascular endothelial cells, components of extracellular matrix (ECM), etc., where immunosuppressive cells play a significant role in tumor immune escape (Li et al., 2021). Increased numbers of CD4^+^CD25^+^ Tregs and MDSC were detected in the peripheral blood of BC patients (Jiang et al., 2023). Tregs mainly exert their immunosuppressive effects by inhibiting CTL proliferation/activation, inhibiting Th1 cytokine secretion, blocking anti-tumor immunity, and inducing MDSC recruitment. Because of its immunosuppressive activity, tumor-invading MDSC can inhibit DC maturation and modulate T-cell activation (Ren et al., 2023). Among the stromal components, CAFs appear to play another key role in BC. Immune evasion could occur through the secretion of soluble immunomodulatory factors, such as IL-1 and TGF-β, by CAF. Around 80% of BC stromal fibroblasts display features of CAFs (Hu et al., 2022). Additionally, a reduction in both number and function (lower IL-12 production and reduced surface expression of HLA-II and CD80/CD86 molecules) was observed when examining DCs isolated from PBMCs and SLMNCs of BC patients (Acikgoz et al., 2022). Metastasis of BC to the tumor-draining lymph nodes is associated with DC apoptosis, maturation block, and inability to form conjugates with CD8^+^ T lymphocytes, rendering them incapable of efficiently priming CTLs against tumor cells (Tharp et al., 2024). Lastly, in BC preclinical models, it has recently been shown that IL-4-producing CD4^+^ T cells directly regulate the behavior of TAMs *in vivo* (Ohno et al., 2021): macrophages can be further divided based on their functional characteristics into classic (M1) or alternative (M2)-activated macrophage subsets. While M1 macrophages are implicated in efficient Ag presentation, induction of Th1 responses, and release of pro-inflammatory cytokines, M2 macrophages secrete low levels of pro-inflammatory cytokines but higher levels of immunosuppressive IL-10. In the majority of human cancers, TAMs acquire an M2 phenotype, creating a permissive environment for tumor growth and dissemination (Nasir et al., 2023); high TAM density is associated with poor prognosis in human BC.

#### 3.2.2 Production of immunosuppressive cytokines

In the tumor immune microenvironment, multiple kinds of intracellular, membrane-bound, or secreted inhibitory molecules can be produced both by tumor cells themselves as well as stromal cells and invading immune cells, mainly including:① immunosuppressants (IL-10, IL-6, IL-4, IL-13, TGF-β, PGE-2, MHC); ② co-inhibitory signals and pro-apoptosis molecules (B7-H1/PD-L1, CTLA-4, TRAIL, FasL); ③ chemokine (CCL2, CCL19, CCL20, CCL21); ④ metabolic enzyme (iNOS, IDO, NADPH oxidase, arginase); ⑤ angiogenic growth factor (VEGF, PDGF-BB, GF). These inhibitory molecules, together with the above stromal cells, constitute a stable immune environment in the tumor microenvironment, which helps evade immune surveillance and promote tumorigenesis (Leonard and Lin, 2023; DePeaux et al., 2023).

Some cytokines and chemokines produced by cancer cells might affect the maturation/or the antitumor effect of some immune cells: increased plasma level of VEGF in BC patients has been associated with lower numbers and maturation degree of DCs (DePeaux et al., 2023). The mechanisms responsible for such alteration may involve increased levels of IL-10 in the sera, which impair DC function by preventing maturation and inducing spontaneous apoptosis (DePeaux et al., 2023). Moreover, IL-10 has been shown to modulate the development of tumor-specific Th1 cells negatively, macrophage production of IL-12, and Ag presentation (Acikgoz et al., 2022). Additionally, BC cells generally overproduce TGF−β; high levels of TGF−β were actually detected in the circulation of BC patients (Acikgoz et al., 2022). TGF−β upregulates the macrophage synthesis of IL−10 and shifts Th1/Th2 balance toward an anti-inflammatory/protumoral Th2 response (Acikgoz et al., 2022). Therefore, it has been suggested that high levels of TGF−β are associated with BC progression and/or lack of response to immunotherapy in BC patients (Acikgoz et al., 2022). BCs also release sMICAs proteins that bind to the activated form of NKG2D receptor and downregulate the cytotoxic activity of NK cells (Wang C. et al., 2025). Finally, BC cells escape killing from NK cells because they have lost the expression of co-stimulatory molecules (B7 family members: B7H1/B7-1/CD80, B7DC/B7-2/CD86, CD40, and CD70), which do not allow full activation of NK cells (Acikgoz et al., 2022): lastly, immunomodulatory cytokines produced by the neoplastic cells themselves (TGF−β) might directly block the activation/functionality of NK cells (Acikgoz et al., 2022). Finally, most human cancers express very high levels of galectin-1 (Gal1) and IDO: first, Gal-1 blocks the effector functions against tumor target cells against tumor target cells and increases the number of immunosuppressive Tregs (Mariño et al., 2023). Gal−1 has recently been identified as one of the main proteins present in breast tumors, positively correlating with the aggressiveness/metastatic potential of BC due to its promotion of a protumoral Th2 response and enlargement of the CD4^+^CD25+Foxp3+ Treg population (Basak et al., 2025). Secondly, IDO causes G0/G1 cell-cycle arrest in alloreactive T-cells (Edwards et al., 2021); actually, higher expression levels of both IDO and Tregs were observed in the sentinel lymph node metastasis of certain BCs (Edwards et al., 2021). The aforementioned immune evasion mechanisms do not operate in a vacuum; they are precisely regulated at the molecular level. Here, we synthesize the two preceding themes, detailing how the ubiquitin system serves as a central switchboard, directly regulating the key pillars of immune evasion: antigen presentation, checkpoint expression, the TME, and immune cell function.

## 4 Ubiquitination-mediated regulation of immune evasion in BC

### 4.1 Ubiquitination in tumor cell-intrinsic immune evasion

Breast cancer cells directly manipulate their own surface molecules and signaling pathways to avoid immune detection and destruction. This section examines how ubiquitination post-translationally controls the key executors of these cell-autonomous evasion strategies.

#### 4.1.1 Regulation of antigen presentation machinery

Building on the general role of ubiquitination in immune evasion, we first examine its specific impact on the initial step of anti-tumor immunity: the presentation of tumor antigens by MHC-I molecules. According to related research reports, activation of deubiquitination can restrict MHC-I antigen presentation and benefit tumor immune escape (Sheng et al., 2021); in BC, it may decrease the recognition and attack of tumor cells by the immune system because of its effect on MHC-I expression/function, thereby promoting tumorigenesis. USP8 regulates MHC-I expression via the TGF-β/SMAD signaling pathway; a USP8 inhibitor could promote T cell-mediated immune responses, increase the expression level of MHC-I, and enhance the sensitivity of tumor cells to immunosurveillance (Yan et al., 2025). Another study shows that USP8 inhibitors can also induce the activation of the innate immune response by regulating TRAF6-NF-κB signaling activity, type-I IFN-signaling, and MHC-I expression (Xiong et al., 2022). Recently, using MS combined with biochemical methods, we identified another E3 ubiquitin ligase, SIAH2, and the DUB OTUD5 as two antagonistic regulators of DBC1 ubiquitination in BC cells. Mechanism exploration reveals that under hypoxic conditions, OTUD5 binding to DBC1 decreases, while SIAH2 competes with DBC1 binding and catalyzes ubiquitination at K287 sites, leading to DBC1 ubiquitination-dependent degradation and affecting BC progression (Liu et al., 2022).

#### 4.1.2 Regulation of Immune Checkpoint Expression

Beyond antigen presentation, the ubiquitin system exerts critical control over immune checkpoint molecules, particularly PD-L1, thereby directly influencing the effector phase of the immune response. According to the latest research reports, TRAF6, previously thought to be a positive and negative regulator of immune cell signal transduction, is now known to the an E3 ubiquitin ligase with a RING finger domain. After the corresponding receptor recognizes PD-L1, USP8 can regulate the degradation of PD-L1 through the removal of K63-connected polyubiquitin chains. At the same time, inhibition of USP8 can counteract K48-connected polyubiquitination and degradation of PD-L1 by promoting the K63-connected polyubiquitination mediated by TRAF6, thereby increasing the expression level of PD-L1 (Xiong et al., 2022). The post-translational modifier enzyme transglutaminase 2 (TG2) was found to promote the ubiquitin–proteasome–mediated degradation of two well-known tumor suppressors (PTEN and IκBα), respectively, and to activate the PI3K/AKT and NF-κB pathways, resulting in increased transcription of CCL2 and PD-L1. [60] Inhibition of TG2 could rescue T cell-dependent cytotoxicity in PD-L1+TNBC cells by downregulating PD-L1 and CCL2 expression (Choi et al., 2020). Another study showed that VGLL4 negatively regulates PD-L1 mRNA levels by inhibiting STAT3 phosphorylation and sensitizes TNBC cells to anti-PD-L1 immunotherapy treatment. Meanwhile, USP15, which acts as a DUB for VGLL4, is also involved in the regulating PD-1 signaling: USP15 deletion causes hyperactivation of IFN-γ-producing T cells, upregulation of PD-L1 and CXCL12, accumulation of Tregs and MDSCs, and the establishment of an immunosuppressive microenvironment (Zou et al., 2015). Recently, PLAC8 was also reported to affect cell proliferation and immune response by modifying the ubiquitination status of PD-L1 (Mao et al., 2022).

### 4.2 Ubiquitination in remodeling the immunosuppressive tumor microenvironment

The tumor microenvironment (TME) is a complex ecosystem that breast cancer cells co-opt to suppress immunity. Here, we explore how ubiquitination modifies both the cellular components and the soluble factors within the TME to foster an immunosuppressive niche.

#### 4.2.1 Regulation of immunosuppressive cells

The cellular composition and functional state of immune cells within the TME are critical determinants of anti-tumor immunity. Ubiquitination serves as a key regulatory layer that orchestrates the differentiation, polarization, and effector functions of both immunosuppressive and cytotoxic immune cells, thereby shaping the overall immune landscape in BC.

Tumor-associated macrophages (TAMs) are highly plastic cells whose polarization towards the immunosuppressive M2 phenotype is a hallmark of BC progression. The ubiquitin system plays a pivotal role in this process. For instance, TRAF3 has been identified as a crucial regulator of macrophage polarization. In addition, we found that TRAF3 increased M1 markers (iNOS, FGR, and SLC4A7) but reduced M2 markers (CD206, CD36, and ABCC3) in macrophages; conversely, TRAF3-deficient cells enhanced LPS-induced M1 polarization but blocked IL-4-induced macrophage polarization. Mechanistically, we revealed here that TRAF3 mainly regulates macrophage polarization through controlling STAT6 K450 ubiquitination (Shi J. H. et al., 2023). This precise control over the STAT6 signaling node exemplifies how ubiquitination can dictate the functional fate of macrophages in the TME.

Tregs are central mediators of immune suppression in BC, and their suppressive capacity is tightly linked to the expression level of the master transcription factor Foxp3. The stability of Foxp3 is dynamically regulated by ubiquitination. In addition, USP7 knockdown in Tregs led to a decrease in the level of Foxp3 protein and the ability of Tregs to suppress effector T cells (van Loosdregt et al., 2013). USP7-mediated deubiquitinase activity was responsible for the stabilization of Foxp3 and Tip60 proteins and the promotion of both Treg immune suppression and tumor growth (Wang et al., 2017). This highlights DUBs like USP7 as key molecular switches that reinforce the immunosuppressive TME by maintaining Treg potency.

Beyond shaping immunosuppressive cells, the ubiquitin system also directly or indirectly impairs the function of effector immune cells, such as T cells and natural killer (NK) cells. BC cells can exploit ubiquitination pathways to evade cytotoxic attack. Recently, Chen X et al. (Chen et al., 2024a) found that the TRIM21 E3 ubiquitin ligase promoted CCT2 ubiquitination and degradation, thereby reversing CCT2’s role in promoting cell transformation. Interestingly, researchers further revealed that exosomes released by BC cells expressing CCT2 could inhibit T lymphocyte activation and pro-inflammatory cytokine production. Notably, the ubiquitin-regulatory function of TRIM21 is not confined to breast cancer. In colorectal cancer, TRIM21 has been found to degrade insulin-like growth factor 2 mRNA-binding protein 2 (IGF2BP2) via the K48-linked polyubiquitination pathway, thereby modulating the stability of the downstream transcription factor FOXM1 and consequently regulating the proliferative and migratory capacities of tumor cells (Bian et al., 2024). This evidence underscores the pivotal role of TRIM21 as an E3 ligase in facilitating immune evasion and tumor progression across multiple cancer types. This represents a novel, exosome-mediated mechanism of immune evasion governed by ubiquitin-dependent protein stability.

#### 4.2.2 Regulation of immunosuppressive soluble factors

Furthermore, the ubiquitin system can modulate key signaling nodes within effector cells. In Tregs, as mentioned, USP7 stabilizes Foxp3. Conversely, in conventional T cells, USP15 deficiency has been shown to cause hyperactivation of IFN-γ-producing T cells, but this is accompanied by an upregulation of PD-L1 and CXCL12, and an accumulation of Tregs and MDSCs, ultimately establishing a net immunosuppressive microenvironment (Zou et al., 2015). This underscores the context-dependent and sometimes paradoxical roles of ubiquitin enzymes in immune regulation.

NK cell function is also susceptible to ubiquitin-mediated regulation. BC cells escape NK cell killing partly by downregulating ligands for activating receptors (such as MICA/B) and co-stimulatory molecules (B7 family members). While the direct ubiquitination of these ligands in BC is an area for further exploration, immunomodulatory cytokines like TGF-β—whose signaling can be modulated by ubiquitination (e.g., via USP8 and the TGF-β/SMAD pathway (Yan et al., 2025))—are known to directly block NK cell activation and functionality (Acikgoz et al., 2022). Additionally, the expression of galectin-1 (Gal-1), a protein that blunts effector T cell function and expands Tregs, is positively correlated with BC aggressiveness. The upstream pathways controlling Gal-1 expression may involve ubiquitin-regulated stability of transcription factors or signaling proteins (Mariño et al., 2023; Basak et al., 2025).

#### 4.2.3 Regulation of signaling networks and key effectors in the tumor microenvironment

The tumor microenvironment (TME) is a fertile ground for immune suppression. This section explores how ubiquitination modifies stromal and immune cells within the TME, such as macrophages and Tregs, to foster an immunosuppressive niche. Overactive ERα signaling has been recognized as one of the significant reasons for luminal BC. The progression of such BCs can usually respond well to the treatment using SERMs (selective estrogen receptor modulators), such as tamoxifen. Very recently, Yang P et al. (Yang et al., 2024) reported that PSMD14 functions as another important DUB in ERα signaling and BC development. It showed from both *in vitro* and *in vivo* studies that PSMD14 stimulated BC progression through ERα signaling, whereas PSMD14 blockade with mercaptopurine (an inhibitor against DUB) abrogated breast tumorigenesis. SENP2 was shown to control ERK2 protein stability by removing SUMO modifications from ERK2, thereby promoting the proliferation and invasion of BC cells and contributing to BC onset/metastasis (Chen et al., 2024b). Notably, we recently uncovered that SMYD4 might act as a tumor suppressor. Here, we demonstrated that SMYD4 suppressed MYH9 binding to CTNNB1 promoter regions by promoting MYH9 K168 monomethylation/ubiquitination degradation (Yang et al., 2025).

### 4.3 Regulation of immune cell function

**FIGURE 2:**
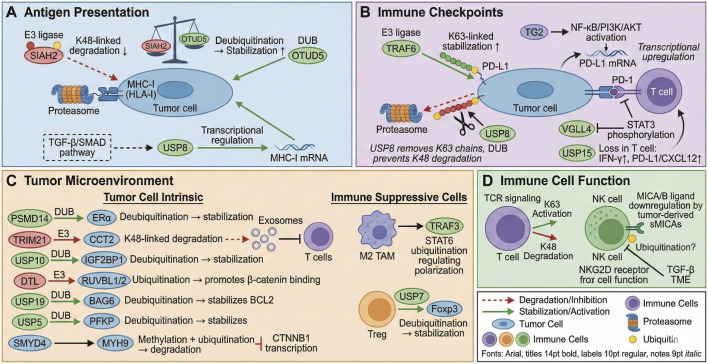
Protein ubiquitination is involved in breast cancer immune escape by regulating tumor antigen presentation, immune checkpoint molecular expression, tumor microenvironment and immune cell function.

Emerging targets are being identified that bridge tumor suppression and immune cell modulation (Li J. et al., 2025). BC: Substrate receptor of E3 ubiquitin ligase KEAP1 (kelch-like ECH-associated protein). As a newly identified prognostic predictor, it plays an essential regulatory role in BC cell proliferation, apoptosis, and the cell cycle (Han et al., 2024). DCAF13 (DDB1 and CUL4-associated factor 13) is another newly discovered E3 ubiquitin CRL4 substrate receptor family member. It is a member of a highly conserved protein family. Research shows that DCAF13 deficiency induces BC cell apoptosis and senescence, and causes G1/S phase BC cell cycle arrest (Shan et al., 2022). High-level expression of MANF was found to be related to poor prognosis of BC patients. When glucose is lacking, SENP1-mediated MANF deubiquitination would prevent the nuclear translocation of MANF and result in an accumulation of cytoplasmic MANF (Xiong et al., 2025). Using co-immunoprecipitation in our lab, we first confirmed that USP10 directly interacts with IGF2BP1 and stabilizes it through deubiquitylation, thereby promoting the invasive and migratory capacity of BC cells (Shi J. et al., 2023). On the other hand, DTL ubiquitylation of RUVBL1 was found to promote the formation of RUVBL1/2-β-catenin complexes, regulate transcriptional levels of DNA NHEJ repair-related genes, and thereby increase radiation resistance in BC (Tian et al., 2024). Both *in vitro* and *in vivo* experiments showed that USP19 could negatively regulate the growth rate of TNBC cells, and positively regulate the ratio of cell apoptosis by regulating BAG6/BCL2 stability (Zhang X. et al., 2023). In addition, USP5-mediated deubiquitination and stabilization of PFKP is required for aerobic glycolysis and TNBC progression (Peng et al., 2024) (Figure 2). The mechanistic insights linking ubiquitination to immune evasion immediately translate into therapeutic opportunities. This chapter reviews the current pharmacological arsenal aimed at the ubiquitin system, including protease inhibitors, ligase inhibitors, and innovative degradation technologies, evaluating their potential to reverse immune evasion in BC.

## 5 Therapeutic strategies targeting protein ubiquitination in BC

The recognition of the ubiquitin-proteasome system as a master regulator of immune evasion has propelled it to the forefront of therapeutic discovery in breast cancer. Moving beyond the broad-spectrum proteasome inhibitor bortezomib, current research is focused on developing agents with superior specificity to target individual components of the ubiquitination machinery, particularly E3 ligases and deubiquitinating enzymes (DUBs). This shift aims to achieve precise modulation of oncogenic or immunosuppressive pathways while minimizing off-target effects. The therapeutic arsenal under investigation is diverse, encompassing small-molecule inhibitors against specific DUBs and E3 ligases, as well as groundbreaking proteolysis-targeting chimera (PROTAC) technology that hijacks the endogenous ubiquitin system for targeted protein degradation. While the field is rapidly evolving with promising preclinical data, the clinical translation of most ubiquitin-targeted agents in BC remains in early stages (Phase I/II), presenting both significant opportunities and challenges for overcoming therapy resistance and improving patient outcomes. This chapter will critically review these major therapeutic modalities, their mechanisms of action, and their current status in the BC therapeutic landscape.

### 5.1 Ubiquitin-specific protease inhibitors

Many ubiquitin-specific protease inhibitors against BC have been reported. Some of them exhibit promising synergistic effects in combination with immunotherapy drugs: USP1 inhibitors such as trifluridine, rottlerin, ML323, etc., all showed excellent anti-tumor effects; trifluridine could inhibit the growth and brain metastasis of TNBC tumors via induction of cell G0/G1-phase arrest and apoptosis (Feng et al., 2018); rottlerin promoted autophagy-mediated apoptosis of BC stem cells and might also possess an anti-angiogenic effect on BC cells (Kumar et al., 2013; Yin et al., 2016). Costunolide, another USP7 inhibitor, suppressed BC by modulating the cell cycle and inducing apoptosis of BC cells in metastatic BC patient-derived xenograft models (Roy and Manikkam, 2015); dehydrocostus lactone showed a synergistic effect (Gao et al., 2023); the USP14 inhibitor b-AP15 displayed synergistic effects when used in combination with either ascorbic acid or anti-PD-L1 antibody for treating TNBCs, and it might be able to combine with crizotinib to treat BC (Pal and Donato, 2014); USP14 inhibitor treatment sensitized BRCA1-mutant and PARPi-resistant TNBC cell lines to PARG inhibitors (Li et al., 2024); USP8 inhibitor increased the levels of PD-L1 expression to elicit immune response and antigen presentation, and may become a new kind of immunoadjuvant drug combined with immunotherapy drugs for controlling tumor growth.

### 5.2 E3 ubiquitin ligase inhibitors

Recently, many studies have demonstrated the rapid development of E3 ubiquitin ligase inhibitors for anti-tumor effects on cell proliferation, invasion, and migration by modulating the function of E3 ubiquitin ligases (Wang et al., 2021). As mentioned above, NEDD4 regulates cell cycle progression of BC cells by down-regulating the ERα expression level; low NEDD4 level predicts sensitivity to endocrine therapies (AI or TAM) treatment and better OS time in BC (Natori et al., 2023). YM155 is the first NEDD4L small-molecule inhibitor with potent anti-tumor activity against BC, achieved by down-regulating surviving (Li X. et al., 2025). Pevonedistat (MLN4924), as the first-in-class NAE inhibitor, can block CRL E3 ligase activation by stabilizing CRL substrate proteins from proteasomal degradation through NAE-mediated inhibition. Although it has demonstrated anti-tumorigenic effects in some cancers (NHL and AML) (Torka et al., 2023), its effectiveness in BC therapy remains under investigation.

### 5.3 PROTAC

PROTAC (PROteolysis TArgeting Chimera) technology is a protein degradation technology based on the UPS (ubiquitin-proteasome system). By designing bispecific molecules to “recruit” E3 ubiquitin ligases to target proteins (POIs), the ubiquitination and degradation of target proteins were induced. Compared with the classical small-molecule inhibitors, the PROTAC technology has better target selectivity and stronger degradation effect (Tong et al., 2024). dBET6 is one kind of BRD4-degrading PROTAC molecule. dBET6 could degrade BRD4 and inhibit the proliferation of tumor cells, and regulate the immune microenvironment of tumors. Researchers have developed a “multifunctional” PROTAC-PDT nanoparticle (dBET6@CFMPD) drug delivery system for the treatment of BC and metastasis. Under the stimulation of MMP-2 in TME (tumor microenvironment), this nanoparticle can release photosensitizer Ce6 and dBET6. The ROS generated by Ce6 will kill cancer cells, while dBET6 degrades BRD4, inhibits the proliferation of tumor cells, downregulates the expression level of PD-L1, and remodels the immunomicroenvironment of the tumor (Tong et al., 2024). ARV-110 is the first PROTAC protein degrader ever developed. After recruiting the E3 ubiquitin ligase, it directly induces ubiquitination and subsequent proteasomal degradation of the androgen receptor (AR), and compared with existing small-molecule drugs, this direct degradation mechanism offers greater advantages for AR degradation (Gough et al., 2024). ARV-471 (Vepdegestrant) is an oral ER PROTAC degrader for the treatment of ER+/HER2− BC. It indirectly induces ubiquitination and subsequent proteasomal degradation of ERs after recruiting the E3 ubiquitin ligase. ARV-471 showed high degradation efficiency against both WT ER and ER mutants (e.g., Y537S/D538G). In the MCF7 xenograft model, the Tumor Growth Inhibition (TGI) values were 85%–120% in the ARV-471 groups, which were higher than those in the fulvestrant group (Gough et al., 2024). In a phase I/II clinical trial, the single-agent treatment with ARV-471 showed good tolerability and promising anti-tumor activity, especially in the ER+/HER2− advanced BC patients who had received prior CDK4/6 inhibitor and endocrine therapy (Hamilton et al., 2024). Combined therapy with a CDK4/6 inhibitor (Palbociclib), an mTOR inhibitor (everolimus), and a PI3K inhibitor (Alpelisib) showed much more powerful anti-tumor effects than monotherapy (Li J. et al., 2025; Gough et al., 2024). In addition, D-PROTAC is another type of “PROTAC” targeting the “undruggable” target STAT3. D-PROTAC binds to STAT3 via DNA decoy and recruits the E3 ubiquitin ligase VHL to degrade STAT3 (Li S. et al., 2025), but whether D-PROTAC has a therapeutic effect on BC still needs further investigation. In conclusion, this review has systematically traversed from the molecular basics of ubiquitination to its complex role in BC immune evasion and the resulting therapeutic strategies. To conclude, we summarize the overarching principles, address current challenges, and propose future directions for both research and clinical translation in this dynamic field.

## 6 Summary and prospects

Ubiquitination modification, as an essential kind of PTM modification that is dynamically and reversibly modified under the synergistic effects of E1–E2–E3 and DUBs, can regulate the protein stability, location, and activity, and is involved in many important cell biological processes, like cell cycle, DNA damage repair, immune response, etc. In TME, abnormal ubiquitination modification levels would lead to the abnormal expression of specific immune checkpoint molecules (PD-L1), antigen presentation-related proteins (MHC class I), and inflammatory signal pathways (NF-κB), promoting tumor immune evasion. Against the background of the current stage of BC treatment with individualized medical development, taking targeted protein modifications into account has emerged as a new concept to improve the efficacy of anticancer drugs. From this perspective, we review the most recent advances in understanding how ubiquitination regulates the formation and development of the BC immune microenvironment and the immune response by modulating its various substrates. As another “master switch” that controls the immune cell’s functions after signal transmission, ubiquitination modification is involved in many immunological key events, such as T cell maturation and development, immune cell differentiation and polarization, and even the last regulatory step was extended to the synthesis/degradation of related molecules such as PD-L1, influencing the response rate of immunotherapy drugs. Different types of ubiquitination modifications were correlated with immune evasion characteristics/drug resistance, respectively, reflecting its “two-sided sword” effect: inducing tumorigenesis on the one hand and restricting anti-tumor therapies on the other. Based on the aforementioned findings, we believe it is necessary to investigate further how ubiquitination regulates the formation of the BC immune microenvironment to provide theoretical support for the clinical application of ubiquitin pathway inhibitors in BC. And the joint administration of ubiquitination-related inhibitor drugs combined with common/new anticancer drugs may achieve better effects on BC patients. Thus, further exploration of ubiquitination modification would benefit from identifying more efficient drugs against BC.

To bridge the gap between mechanistic studies and clinical application, future research must leverage large-scale multi-omics datasets from breast cancer patients. Systematic bioinformatics analysis of public repositories (e.g., TCGA, METABRIC) can be employed to evaluate the expression profiles of key E3 ligases and DUBs discussed herein. Crucially, their correlation with established immune parameters—such as tumor-infiltrating lymphocyte (TIL) density, immune gene expression signatures (e.g., IFN-γ response), and estimates of immunosuppressive cell abundance—should be rigorously assessed. Furthermore, determining whether the expression of specific ubiquitin-system components predicts patient prognosis, intrinsic subtypes, or response to immunotherapy (e.g., in cohorts from IMpassion130 or KEYNOTE trials) will be invaluable. Such analyses can distinguish *bona fide* drivers of immune evasion with clinical relevance from context-dependent modifiers, thereby prioritizing the most promising therapeutic targets for functional validation and drug development.

## Glossary

APM: Antigen Processing Machinery
BC: Breast Cancer
CAF: C-C Motif Chemokine Ligand
CCL: C-C Motif Chemokine Ligand
CDK: Cyclin-Dependent Kinase
CTL: Cytotoxic T Lymphocyte
CTLA-4: Cytotoxic T-Lymphocyte-Associated Protein 4
DUB: Deubiquitinating Enzyme
ECM: Extracellular Matrix
ER: Estrogen Receptor
HER2: Human Epidermal Growth Factor Receptor 2
HLA: Human Leukocyte Antigen
IDC: Invasive Ductal Carcinoma
IDO: Indoleamine 2,3-Dioxygenase
IFN: Interferon
IL: Interleukin
ILC: Invasive Lobular Carcinoma
MDSC: Myeloid-Derived Suppressor Cell
MHC: Major Histocompatibility Complex
NF-κB: Nuclear Factor Kappa-B
NK cell: Natural Killer cell
PD-1: Programmed Cell Death Protein 1
PD-L1: Programmed Death-Ligand 1
PROTAC: Proteolysis-Targeting Chimera
PTM: Post-Translational Modification
RBR: Ring-Between-Ring
RING: Really Interesting New Gene
STAT: Signal Transducer and Activator of Transcription
TAM: Tumor-Associated Macrophage
TCR: T Cell Receptor
TGF-β: Transforming Growth Factor-Beta
Th: T Helper Cell
TIGIT: T Cell Immunoreceptor with Ig and ITIM Domains
TIM-3: T-Cell Immunoglobulin and Mucin-Domain Containing-3
TLR: Toll-Like Receptor
TME: Tumor Microenvironment
TNBC: Triple-Negative Breast Cancer
TNF: Tumor Necrosis Factor
Treg: Regulatory T Cell
UPS: Ubiquitin-Proteasome System
USP: Ubiquitin-Specific Protease
VEGF: Vascular Endothelial Growth Factor
